# Developmental changes in shortening of pro-saccade reaction time while maintaining neck flexion position

**DOI:** 10.1186/s40101-017-0161-7

**Published:** 2018-01-10

**Authors:** Kenji Kunita, Katsuo Fujiwara, Naoe Kiyota, Chie Yaguchi, Takeo Kiyota

**Affiliations:** 1 Department of Sports Instruction, Faculty of Sports and Human, Sapporo International University, 4-1-4-1 Kiyota, Kiyota-ku, Sapporo, 004-8602 Japan; 2 Department of Sports and Health, Kanazawa Gakuin University, 10 Sue-machi, Kanazawa, 920-1392 Japan; 3 Department of Rehabilitation, Japan Health Care College, 6-17-3 Megumino-nishi, Eniwa, 061-1373 Japan

**Keywords:** Development, Pro-saccade reaction time, Brain activation, Neck flexion

## Abstract

**Background:**

We investigated developmental changes in shortening of pro-saccade reaction time while maintaining neck flexion.

**Methods:**

Subjects comprised 135 children (3–14 years) and 29 young adults (19–23 years). Children were divided into six groups in 2-year age strata. Pro-saccade reaction tasks for 30 s were performed in neck rest and flexion positions. Reaction times under each position were averaged in every 10-s period.

**Results:**

Under neck rest position, reaction time in the 0–10 s period was significantly longer in the 3- to 4-year-old group than in the 5- to 6-year-old group and above. No significant age effect was found for reaction time in the 0–10 s period in the 5- to 6-year-old group and above. Although a significant effect of neck flexion was not observed until the 9- to 10-year-old group, significant shortening of reaction time with neck flexion was found in the 11- to 12-year-old group and above. Furthermore, this shortening was maintained until the first 20–s period in the 11- to 12-year-old group and during the entire 30 s in the 13- to 14-year-old and above.

**Conclusions:**

These results suggest that brain activation with the maintenance of neck flexion, related to shortening of the pro-saccade reaction time, was found from a later age of approximately 11 years and above, compared with the age at which information-processing function in the pro-saccade was enhanced. In addition, brain activation with the maintenance of neck flexion was sustained longer with age.

## Background

In daily life, saccadic eye movements are often used to quickly and precisely shift the gaze to an appearing visual target. Such eye movement is called visually guided saccade, or pro-saccade. The pro-saccade is controlled only via the supraspinal pathways, including the lateral genicular body, occipital cortex, posterior parietal cortex, parietal eye field, frontal eye field, superior colliculus, and reticular formation [1–5]. Information-processing time in the neural pathway for the pro-saccade has been investigated using the reaction time method. Pro-saccade reaction time changes with development, shortening from early childhood to approximately 6-8 years old, then reaching a similar level to that in young adults [6–13]. One of the neurological factors causing these age-related changes in childhood is probably myelination in the neural pathway of pro-saccade [8, 14].

The saccade system is also reportedly supported and/or promoted by the brain activation system [15–18]. We have previously reported that maintaining a flexed neck position, which constitutes part of the dynamic posture [19], leads to shortening of the pro-saccade reaction time [20, 21]. This shortening was observed without the chin resting on a stand, so the neck extensors would have been activated [20]. We also reported that pro-saccade reaction time shortened with voluntary isometric contraction of the superficial neck extensors by shoulder girdle elevation [22], or with vibration stimulation to the trapezius, one of the superficial neck extensors [23, 24]. These findings suggest that the shortening with maintaining neck flexion posture is attributable to an ascending brain activation system in tandem with increases in muscle afferent information [25–27]. The following changes in other physiological indices also support brain activation during neck flexion: 1) shortening of limb reaction times and latencies of visual, auditory and somatosensory evoked potentials [28, 29]; 2) increased amplitudes of auditory evoked potentials and event-related potentials associated with motor preparation and cognition [28–30]; and 3) shortened latencies and increased amplitudes of motor evoked potentials evoked by transcranial magnetic stimulation [31].

We have demonstrated that such shortening of the reaction time was affected by training [32, 33] and aging [33, 34], suggesting that the brain activation seen with the maintenance of neck flexion may be an acquired factor. However, when such brain activation function is acquired within the period from childhood to adolescence has not yet been reported. The ascending activation system is known to consist of two subsystems: a dorsal pathway from the reticular formation to the thalamus and prefrontal cortex; and a ventral pathway from the reticular formation to the hypothalamus and prefrontal cortex [35, 36]. Myelination in the reticular formation and frontal lobe, contributing to brain activation, is continuously observed after 10 years old [37–40]. Furthermore, developmental improvement of the performance related to frontal lobe function is observed until a later age, compared with that of pro-saccade performance [41, 42]. Considering these developmental changes, the appearance of the brain activation with the maintenance of neck flexion would be found from a later age, compared with the age when the information-processing function in the pro-saccade enhances.

Furthermore, brain activation in young adults with the maintenance of a neck flexion position, related to the shortening of the pro-saccade reaction time, is reportedly maintained for around 30 s [43]. However, developmental changes in the continuity of brain activation have never been investigated. The continuity of brain activation might be related to sustained attentional function [43]. The function of sustained attention to maintain early response to a visual target has been suggested to enhance from infant to young adulthood [44, 45]. Considering these findings, brain activation with neck flexion would be sustained longer with age.

The present study investigated developmental changes in shortening of pro-saccade reaction time while maintaining the neck flexion position and the continuity of this shortening. The working hypotheses were as follows. First, the shortening of the pro-saccade reaction time with neck flexion would be found from a later age, compared with the age at which the pro-saccade reaction time decreases. Second, this shortening of reaction time during neck flexion would be sustained longer with age.

## Methods

### Subjects

Subjects comprised 135 children (age range, 3-14 years), and 29 young adults (age range, 19-23 years). Children were divided into 6 groups by every 2-year-old: 13 children in the 3- to 4-year-old group; 31 in the 5- to 6-year-old group; 18 in the 7- to 8-year-old group; 24 in the 9- to 10-year-old group; 21 in the 11- to 12-year-old group and 28 in the 13- to 14-year-old group. No subjects had any history of neurological impairment. In accordance with the Declaration of Helsinki, all parents and young adults provided written informed consent to participate in this study after receiving an explanation of the experimental protocols and how privacy would be protected. All experimental protocols were approved by our institutional ethics committee.

### Apparatus and data recording

**Fig. 1:**
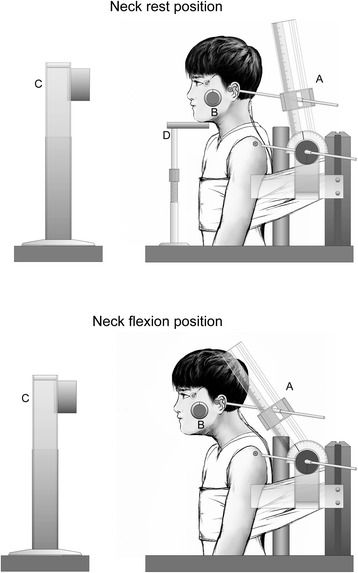
Experimental setup. A: Neck angular detector. B: Head inclination angular detector using the pendulum principle. C: Visual stimulator. D: Chin stand

The experimental setup is shown in Fig. 1. Subjects sat on a steel-frame chair with the back resting against a vertical wall, and the trunk was secured by a cotton band to prevent antero-posterior movement. The knees were kept flexed at approximately 90° and the feet were rested on a low table. Neck flexion angle was measured as described by Fujiwara et al. [20]. The flexion angle was defined as the rotational angle of the tragus around the acromion in the sagittal plane, with the starting position (0°) as the neck rest position. We determined 80% of maximal neck flexion angle (neck flexion position) for each subject using a custom-made angular detector with the center point set at the acromion, while regulating the distance between the acromion and tragus. The set angle in the flexed position was defined as described by Fujiwara et al. and Kunita and Fujiwara [28, 31]. Head inclination angle was determined as the angle between the auriculo-infraorbital line and the gravitational line, and this was maintained at the same angle as the sitting posture to maintain constant sensory inputs from the vestibular organ. An angular detector using the pendulum principle (Level + angle detector; Mitsutomo, Tokyo, Japan) was attached to the temple to confirm this angle. A chin stand was used to support the head and allow maximal relaxation of the neck extensor muscles.

**Fig. 2:**
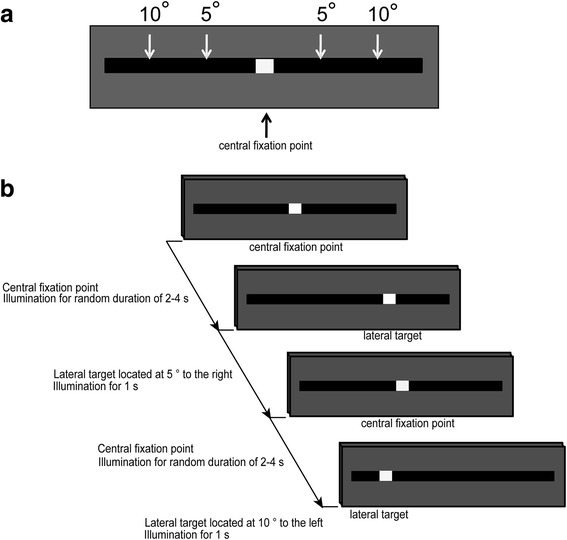
**a** Four lateral targets located at 5° and 10° to the left and right of the central fixation point. **b** Presentation protocol in the pro-saccade task

A visual stimulator with light-emitting diodes (LEDs) (SLE-5100; Nihon Kohden, Tokyo, Japan) was used to induce saccadic eye movement. LEDs were located at the central fixation point and at the targets and were illuminated for time periods set by a functional generator (WF1966; NF, Kanagawa, Japan). The size of the LED was 0.23° in height and 0.80° wide, with illuminance of 0.02 lumen. LEDs were placed at the height of the nose root, and the distance between the LED at the central fixation point and the nose root was set at 50 cm. The central fixation point was illuminated for a random duration of 2–4 s, and one of the lateral targets was subsequently illuminated for 1 s (Fig. 2). The four lateral targets were located at 5° and 10° to the left and right of the central fixation point, and were presented with equal probability at random.

Horizontal eye movements and blinks were measured using electrooculography (EOG). Horizontal EOG was recorded from surface electrodes (P-00-S; Ambu, Ballerup, Denmark) at the outer canthus of each eye, and vertical EOG from electrodes above and below the left eye. A ground electrode was placed at the center of the forehead. Electrode-input impedance was reduced to < 10 kΩ. The signal from the electrodes was amplified (×2000) using a DC amplifier (AN-601G; Nihon Kohden, Tokyo, Japan). To obtain stable EOG traces, recording began at least 20 min after the placement of electrodes. Surface electrodes with bipolar derivation were used to monitor and record surface electromyography (EMG) activity of the bilateral upper trapezius muscles. Inter-electrode distance was about 3 cm. A ground electrode was placed at the spinous process of the C7 vertebra. Electrode impedances were reduced to < 5 kΩ. Signals from these electrodes for trapezius were amplified (×2000) and band-pass filtered (5–500 Hz), using an EMG amplifier (MA1000; DIGITEX, Tokyo, Japan). EOG and visual stimulus data were sent to a computer (Dimension E521; Dell, Kawasaki, Japan) via an A/D converter (ADA16-32/2(CB) F; Contec, Osaka, Japan) at 1000 Hz with 16-bit resolution. EMG data were sent to an oscilloscope (DS-6612; Iwatsu, Tokyo, Japan) to monitor activation.

### Procedure

Pro-saccade reaction task was carried out under neck rest and neck flexion positions. Initially, to familiarize subjects with pro-saccade, 20-s practice trials in the neck rest position were performed three times. Next, 30-s experimental trials were repeated five times in each postural condition. Prior to beginning each trial, contraction and relaxation of the trapezius muscles were alternated several times and deep breaths was taken to relax the muscle. The experimenter verbally instructed the subject to relax the trapezius muscle, with monitored by EMG. After relaxation of the muscle was confirmed, LED illumination was start. Subjects were instructed to turn their gaze as quickly as possible to the illuminated target. In the neck flexion position, the subject first performed neck flexion within approximately 3 s, maintained this position for 2 s, and then started each trial. Neck angles and head inclination angle during the saccadic task were monitored from the left side by an experimenter. Rest periods of 1 min and 3 min were provided between trials and between conditions, respectively. The order of neck position conditions was counterbalanced across subjects to consider potential effects of fatigue and sequential learning.

### Data analysis

**Fig. 3:**
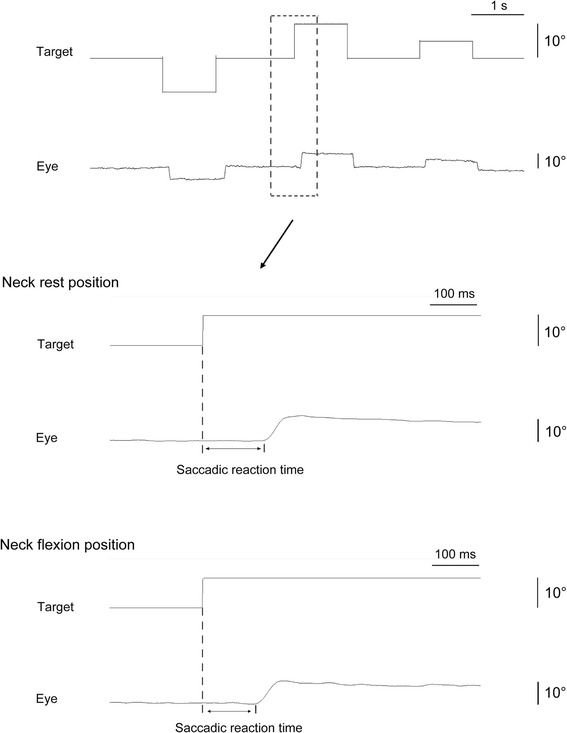
Analysis of saccadic reaction time in two sample records (neck rest position and neck flexion position)

Analysis of visual stimuli, pro-saccade, and EMGs of the trapezius were performed using BIMUTAS-II software (Kissei Comtec, Matsumoto, Japan). Pro-saccade reaction time was defined as the latency between onset of target movement and beginning of eye movement (Fig. 3). Onset of eye movement was determined by visual inspection of EOG displacement that was easily discernible from baseline. The pro-saccade reaction time for each condition was averaged for every 10-s period.

To analyze the activity level of upper trapezius muscles, all EMGs were 40-Hz high-pass filtered using the seventh-order Butterworth method to exclude electrocardiographic and movement artifacts, then full-wave rectified. In each neck position, mean EMG amplitude of each side of the upper trapezius muscle for every 10-s period was calculated. Thereafter, relative ratio of the EMG amplitude of the neck flexion condition against the neck rest condition was calculated using the following equation for each trapezius muscle in each 10-s period:

$$ \mathrm{Relative}\  \mathrm{ratio}=\left(\mathrm{EMGnx}-\mathrm{EMGmin}\right)/\left(\mathrm{EMGrx}-\mathrm{EMGmin}\right) $$

where EMGnx is EMG mean amplitude at the neck flexion position in each of the 15 time periods (5 trials × 3 time periods), EMGmin is the smallest value in mean EMG amplitude at the neck rest position, and EMGrx is the mean EMG amplitude at the neck rest position in each of the 15 time periods. After that, relative ratios from right and left sides in 5 trials were averaged separately for each 10-s period.

### Statistical analysis

Data were tested for normality using the Shapiro-Wilks test and for equality of variance using Leven’s test. Two-way analysis of variance (ANOVA) was used to assess the effect of age (3- to 14-year-old children and young adults) and time period (0-10 s, 10-20 s and 20-30 s) on relative ratio of EMG amplitude. Two-way repeated-measures ANOVA was used to assess the effect of time period and neck position (neck rest and flexion positions) on saccade reaction time in each age group. When significant main effects of time period and neck position were recognized, the post hoc Tukey’s honestly significant difference (HSD) test was used to investigate differences within the time period and the Bonferroni-adjusted paired t-test was used to investigate differences within neck position, respectively. One-way ANOVA was used to compare reaction times for the 0–10 s period in the neck rest position across age groups. Post hoc comparisons were performed using Tukey’s HSD test to further examine differences suggested by ANOVA. The alpha level was set at *p* < 0.05. All statistical analyses were performed using IBM SPSS Statistics version 21 (IBM Japan, Tokyo, Japan).

## Results

**Fig. 4:**
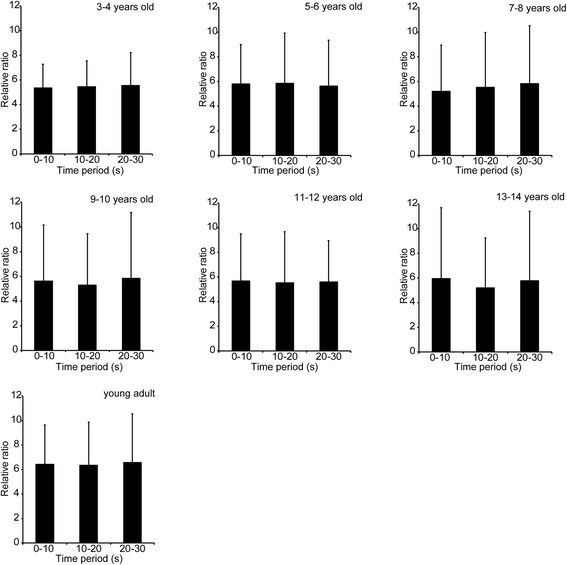
Mean and standard deviation of relative ratio of EMG amplitude in every 10-s time band

Means and standard deviations of relative ratio of EMG mean amplitude in the trapezius are shown in Fig. 4. The relative ratio was within the range from 5.20 to 6.57, and no significant main effect of age or time period, or interaction between these factors was found in the relative ratio.

**Fig. 5:**
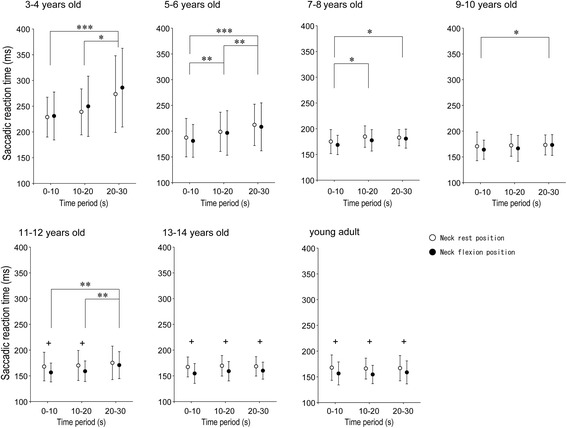
Mean and standard deviation of pro-saccade reaction time at neck rest position and neck flexion position in every 10-s time band. Asterisk indicates significant difference between time bands. * < 0.05; ** < 0.01, *** < 0.001. Plus indicates significant difference between postural conditions. + < 0.01

Means and standard deviations of pro-saccade reaction time in each 10-s period are shown in Fig. 5. For all groups from 3 to 10 years old, only a significant main effect of time period was found in the reaction time (3- to 4-year-old group: *F*_2,24_ = 5.33, *p* < 0.05; 5- to 6-year-old group: *F*_2,60_ = 23.73, *p* < 0.001; 7- to 8-year-old group: *F*_2,34_ = 3.90, *p* < 0.05; 9- to 10-year-old group: *F*_1.60,36.75_ = 3.66, *p* < 0.05). For the 3- to 4-year-old and 5- to 6-year-old groups, reaction time was significantly longer in the order of 20-30 s, 10-20 s and 0-10 s periods (*ps* < 0.05). For the 7- to 8-year-old group, reaction time was significantly longer in the 10–20 s and 20–30 s periods than in the 0–10 s period (*ps* < 0.05). For the 9- to 10-year-old group, reaction time was significantly longer in the 20–30 s period than in the 0–10 s period (*p* < 0.05). For the 11- to 12-year-old group, significant main effects of time period (*F*_1.56,31.16_ = 7.08, *p* < 0.01) and neck position (*F*_1,20_ = 12.12, *p* < 0.01) were found, but no significant interaction was apparent. Although no significant differences in reaction time were found in the neck rest position, reaction time at the neck flexion position was significantly longer in the 20–30 s period than in the other two periods (*ps* < 0.05). In the 0–10 s and 10–20 s periods, reaction time was significantly shorter at the neck flexion position than in the neck rest position (*ps* < 0.05). For the 13- to 14-year-old and young adult groups, the only significant main effect of neck position was found in the reaction time (13- to 14-year-old group: *F*_1,27_ = 43.97, *p* < 0.001; young adults: *F*_1,28_ = 57.44, *p* < 0.001). In every time period, reaction time was significantly shorter in the neck flexion position than in the neck rest position (*ps* < 0.01).

To investigate age-related changes in reaction time at the neck rest position regardless of time-dependent prolongation, we compared reaction times for the 0–10 s period among all age groups. A significant effect of age was found in reaction time at the neck rest position for the 0–10 s period (*F*_6,60.5_ = 5.50, *p* < 0.01). Reaction time was significantly longer in the 3- to 4-year old group than in the 5- to 6-year-old group and above (*ps* < 0.01). No significant differences in reaction time were observed among the 5- to 6-year-old group and above.

## Discussion

Significant time-dependent prolongations in pro-saccade reaction time at the neck rest position were found for all groups from 3 to 10 years old. Age-related changes at the neck rest position were then examined, using the reaction time for the 0–10 s period. This reaction time for the 0–10 s period was significantly longer in the 3- to 4-year-old group than in the 5- to 6-year-old group and above. No significant age difference in reaction time for the 0–10 s period was observed among the 5- to 6-year-old group and above. These results indicate that age-related enhancement of information-processing function in the pro-saccade was observed until at least 5–6 years old, by which the function reached similar levels to young adults, in line with the findings of previous studies [8, 11, 46, 47].

Significant shortening of pro-saccade reaction time with neck flexion position was not found until the 9- to 10-year-old group, but was evident in the 11- to 12-year-old group and above. These findings suggest that brain activation with maintenance of the neck flexion position would be acquired after approximately 11 years old. No significant age effect was found in relative ratio of EMG mean amplitude in the trapezius. This indicated that muscle activity levels of the neck extensors were unrelated to developmental changes in the shortening of pro-saccade reaction time while maintaining neck flexion. Myelination of the brainstem reticular formation and frontal lobe, contributing to brain activation, has been reported to be observed after 10 years old [37–40]. Furthermore, age-related improvements in performances related to frontal lobe function have been suggested to start later than those of pro-saccade performance [41, 42]. Relating to such developmental differences in brain function, the results in the present study suggest that the appearance of brain activation while maintaining a neck flexion position, which causes shortening of the pro-saccade reaction time, was found from a later age, compared with the age when the information-processing function in the pro-saccade enhanced.

The significant shortening of the pro-saccade reaction time with neck flexion was sustained until 20-s period in the 11- to 12-year-old-group and during the entire 30 s in the 13- to 14-year-old and young adult groups. This result suggests that age-related marked enhancement of sustained brain activation with the neck flexion was observed even later, at approximately 13 years and above, compared with the age at which brain activation with neck flexion appeared. The result that no significant effect of time period was found in the relative ratio of EMG amplitude suggests that time-dependent changes in muscle activity of the neck extensors were unrelated to development of sustained brain activation. Such sustained brain activation might be related to attentional function [43]. Sustained attention to maintain early response to appearing visual targets increases from infancy to early adulthood [44, 45]. In particular, sustained attention has been reported to be markedly enhanced after 13-14 years [45]. Considering these findings, marked enhancement of sustained attention after 13 years might be related to sustained brain activation with the maintenance of neck flexion.

## Conclusions

The results in the present study suggested that the brain activation with the maintenance of neck flexion, related to shortening of the pro-saccade reaction time, was found from a later age at approximately 11 years and above, compared with the age at which information-processing function in the pro-saccade enhanced. In addition, brain activation with neck flexion was suggested to be sustained longer with age.

## Abbreviations

ANOVA: Analysis of variance
EMG: Electromyography
EOG: Electrooculography
HSD: Honestly significant difference
LED: Light-emitting diode
